# Innovative Approach to Embolization of Pelvic Varices Using Endovaginal Guidance: Methodology and Early Outcomes †

**DOI:** 10.3390/jpm15100500

**Published:** 2025-10-17

**Authors:** Eva Fourage-Jambon, Rayann Soueidan, Hamza Sawalha, Yassine Lamfichekh, Benjamin Linares, Hugo Hans, Mathieu David, Isabelle Molina-Andreo, Charlotte Douchez, Nicolas Pangon, Yann Le Bras, Rim Maaloum, Clément Marcelin

**Affiliations:** 1 Service d’Imagerie Médicale Adulte, Hopital Pellegrin, CHU de Bordeaux, F-33076 Bordeaux, France; rayann.soueidan@chu-bordeaux.fr (R.S.); yassine.lamfichekh@chu-bordeaux.fr (Y.L.); benjamin.linares@chu-bordeaux.fr (B.L.); h2hans@gmail.com (H.H.); mathieu.david@chu-bordeaux.fr (M.D.); isabelle.molina-andreo@chu-bordeaux.fr (I.M.-A.); charlotte.douchez@chu-bordeaux.fr (C.D.); nicolas.pangon@chu-bordeaux.fr (N.P.); yann.lebras@chu-bordeaux.fr (Y.L.B.); rim.maaloum@chu-bordeaux.fr (R.M.); clement.marcelin@chu-bordeaux.fr (C.M.); 2 ICare Team, Bordeaux Institute of Oncology (BRIC INSERM U1312), University of Bordeaux, F-33076 Bordeaux, France; 3 Department of Vascular and Interventional Radiology, Image-Guided Therapy Center, François-Mitterrand University Hospital, BP 77908, 21000 Dijon, France; sawalhahamza@gmail.com

**Keywords:** embolization, endovaginal, glue, ethylene vinyl alcohol copolymer, efficacy, venous insufficiency, personalized medecine

## Abstract

**Objective:** To retrospectively assess the safety and efficacy of endovaginal guidance for embolizing perivaginal varices associated with persistent localized symptoms, including dyspareunia and postcoital pain. **Methods:** From February 2024 to January 2025, 10 women (median age: 36 years, range: 23–45) underwent pelvic embolization using endovaginal guidance. Eight patients had already undergone endovascular embolization, with persistent perivaginal varices that were inaccessible by this approach, accompanied by dyspareunia or postcoital pain. Primary efficacy was assessed three months post-embolization, defined as a Visual Analog Scale (VAS) score of <2 and a ≥50% decrease. Outcomes were assessed through clinical and imaging follow-up. **Results:** Technical efficacy was achieved in all procedures (100%). Embolization was performed using Glubran/Lipiodol in eight cases (80%) and Onyx^®^ in two cases (20%). The primary efficacy of the procedures was 90.0% (9 out of 10 cases). A reduction in dyspareunia and postcoital pain was observed, with median VAS scores decreasing to one and zero, respectively, compared to initial scores of seven and seven (*p* = 0.002 and *p* = 0.016) and to scores after endovascular embolization to five and five (*p* = 0.004 and *p* = 0.016). No major complications were recorded. Imaging follow-up showed a significant reduction in perivaginal varicosities in all cases. **Conclusions:** Endovaginal guidance proves to be a fast and effective technique for the embolization of perivaginal varices, highlighting its integration into the principles of personalized medicine.

## 1. Introduction

Pelvic congestion syndrome (PCS) is a multifactorial vascular disorder first described in 1949, yet its pathophysiological mechanisms have only been elucidated in recent decades. Patients frequently undergo prolonged diagnostic delays, largely due to the heterogeneity of clinical manifestations and the persistent lack of consensus regarding clinicoradiological correlation. The 2025 French radiological guidelines provide, for the first time, a structured framework for both diagnostic evaluation and endovascular therapeutic management. Among the most prevalent symptoms, dyspareunia and postcoital pain are predominantly attributed to pathological iliac venous tributaries [1].

It is now understood that this venous dilation triggers the release of pain-related hormones. The alteration of friction forces on the endothelium (shear stress) produced by blood flow is another essential factor that can promote local inflammation of the venous wall [2]. In fact, several experimental studies have shown that shear stress, through integrins anchored in the endothelial cell membrane, can influence many intracellular biochemical processes, such as protein G phosphorylation, activation of tyrosine kinases, free radical production, and the synthesis of different nuclear transcription factors [3].

The treatment for these symptoms involves, on the one hand, stopping the reflux that feeds these varices and, on the other hand, occluding the dilated varices themselves [4,5]. Accessing perivaginal varices is often challenging via the endovascular route due to narrowing and the presence of valves originating from the internal iliac veins [6]. For diagnostic purposes, perivaginal varices are clearly visualized using endovaginal ultrasound, appearing as tubular, fluid-filled structures with stagnant flow [7,8]. Therefore, direct puncture of these dilated varices appears to be a relevant option when the varices are inaccessible via the endovascular approach. The aim of this study is to demonstrate the efficacy and safety of this approach in the embolization of perivaginal varices causing localized symptoms such as dyspareunia and postcoital pain.

## 2. Materials and Methods

This was a single-center, retrospective study performed at a university hospital. This retrospective study was approved by the institutional ethics review board (IRB N° CER-BDX 2025-168). Informed consent was obtained for all patients.

### 2.1. Patients

From February 2024 to January 2025, 10 consecutive women (mean age 36 years, range: 23–45) were retrospectively included to undergo embolization of perivaginal varices with endovaginal guidance.

Patients were eligible if they had dilated perivaginal veins with dyspareunia or postcoital pain persisting after initial embolization, or if pre-interventional MRI (T1 FAT SAT Gadolinium sequence) suggested that access to the iliac afferents would be challenging.

Patient characteristics are summarized in Table 1.

Initial symptoms and medical background were recorded during a specialized consultation at the same time as diagnostic imaging by MRI. For 8 patients, a first phlebography and embolization by femoral access was performed. A post-embolization MRI was performed 3 months after endovascular embolization. If MRI showed persistent perivaginal varices and corresponding symptoms such as dyspareunia or postcoital pain, embolization with endovaginal guidance was proposed. For one patient (patient 7), endovascular embolization was not performed due to her nulliparous status, young age, absence of reflux or ovarian vein dilation on MRI, and symptoms limited to perivaginal varicosities, presenting as dyspareunia and postcoital pain. For one patient (patient 9), left ovarian vein embolization was performed simultaneously with embolization under endovaginal guidance.

The following three parameters were retained for the evaluation of perivaginal veins symptoms: “pelvic pain”, “dyspareunia”, “postcoital pain”. The Visual Analog Scale (VAS) between 0 and 10 was recorded for each parameter during the consultation, “initial VAS”, 3 months after endovascular embolization, “post-endovascular VAS”, and 3 months after embolization by endovaginal guidance, “final VAS”.

### 2.2. Procedure

**Figure 1:**
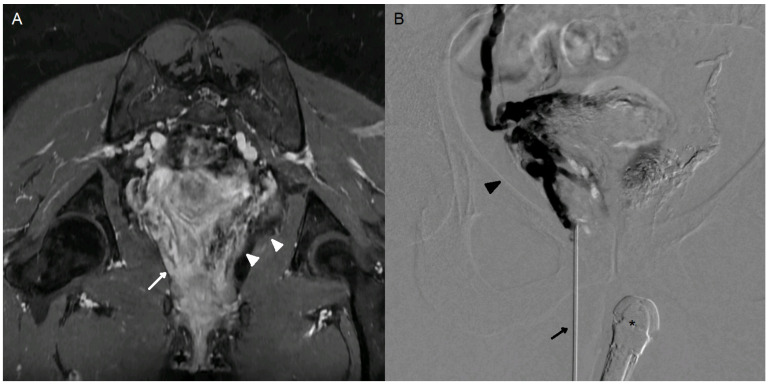
A 35-year-old woman (case n°6), G1P1, presenting with a 3-year history of chronic pelvic pain. Significant improvement in symptoms was achieved following endovascular embolization. However, dyspareunia (VAS 7/10) and postcoital pain (VAS 6/10) persist. (**A**) Oblique coronal MRI in the vaginal axis, LAVA FS sequence following gadolinium-based contrast injection (GE Systems, Signa Hero), demonstrating persistent right perivaginal varices (white arrow). The embolic agent (Onyx) is clearly visualized on this 3D gradient-echo sequence as a susceptibility artifact in the left perivaginal varices (white arrowhead). (**B**) Percutaneous puncture of right perivaginal varices (dark arrowhead) with a n18-gauge needle (dark arrow) under endovaginal guidance (*). Slow injection of iodinated contrast agent demonstrating opacification of the entire perivaginal and peri-uterine variceal network on the right side.

All procedures (*n* = 10) were performed on the same angiographic unit (Artis Pheno, Siemens, Malvern, PA, USA) and US unit (Supersonic, Siemens, Malvern, PA, USA). Embolizations were performed by two investigators with 5 and 8 years’ experience, familiar with a variety of embolic devices and techniques (E.FJ, C.M). Under general anesthesia or sedation (*n* = 8 and *n* = 2, respectively, depending on patient preference), the woman was placed in the frog-leg position. An endovaginal probe was inserted into the vagina to visualize perivaginal varices as tortuous, dilated vessels with stagnant flow. An 18-gauge, 15 cm needle was used to puncture the varices. The endovascular position was confirmed by blood reflux and contrast injection through extension tubing to avoid hand exposure under fluoroscopy. An anterograde phlebography was performed by slow injection, which provided visualization of the entire varicose network and the pathologic afferent vein (Figure 1).

**Figure 2:**
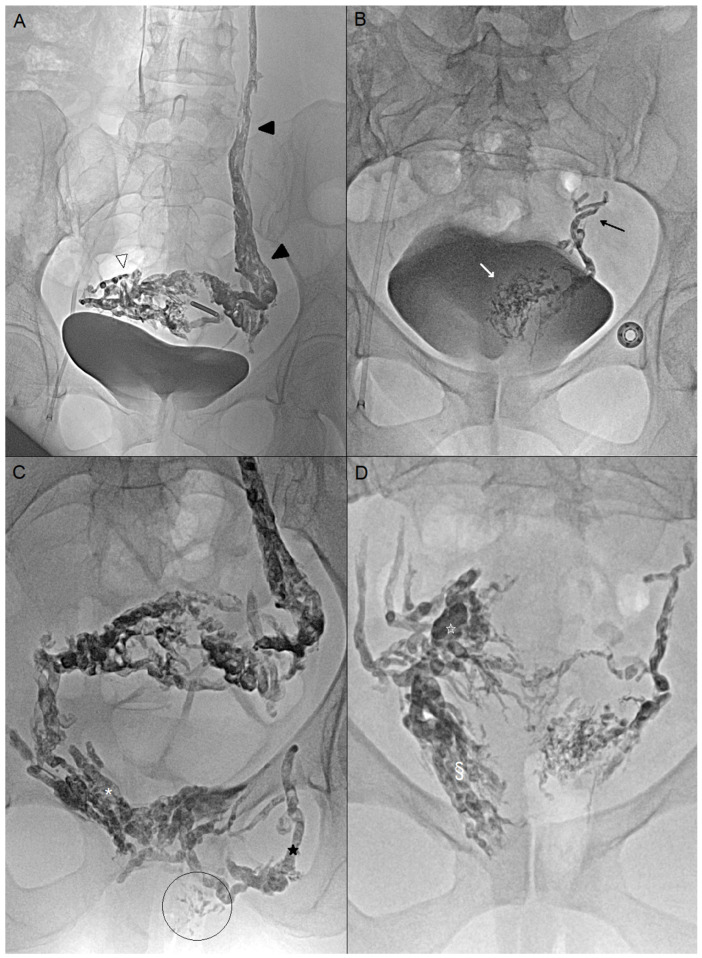
(**A**) Endovascular embolization by femoral access of a 45-year-old woman (case n°4). Embolization of the left ovarian vein (black arrowheads) with Onyx, which also enables occlusion of the right peri-uterine varicose veins (white arrowhead) (**B**) Endovascular embolization of a 35-year-old woman (case n°6). No pathological reflux in the left ovarian vein. Embolization of the left uterine vein (black arrow) and left pericervical varices (white arrow) with Onyx, catheterized using a UAC catheter (Merit Medical, South Jordan, UT, USA) and a Progreat 2.7Fr microcatheter (Terumo, Tokyo, Japan). (**C**) Case n°4: Percutaneous embolization under endovaginal guidance of the bilateral pudendal veins (white asterisk), the left obturator vein (black star), and vulvar varices (circled area). Glue was preferred due to the presence of superficial vulvar varices. (**D**) Case n°6: Percutaneous embolization of right perivaginal (§) and periuterine varices (white star) under endovaginal guidance. Onyx was preferred for its slow and controlled progression.

Then, embolization was performed with a liquid agent. Glue was preferred when certain varicose veins were superficial to avoid permanent tattoos [9,10]. Glubran^®^2 (GEM, Viareggio, Italy) was mixed with Lipiodol^®^ (Guerbet, Paris, France) (ratio 1:1 or 1:2) and injected with 3 mL syringe. At the end of the injection, the needle and superficial varices were flushed with glucose, followed by a 1 min compression of the vulvar puncture site. Ethylene vinyl alcohol copolymer (Onyx^®^) (Medtronic, Dublin, Ireland) was prepared by agitation for at least 20 min. The needle was rinsed and the dead space was plugged with dimethyl sulfoxide (DMSO). Then, Onyx^®^ was delivered with 1 mL syringe provided. Onyx^®^ was slowly injected under fluoroscopic control (Figure 2).

### 2.3. Efficacy

Technical efficacy was considered when varicose veins visualized by endovaginal US were occluded. Primary efficacy was considered when all VAS scores were < 2/10 with a ≥50% decrease three months after embolization. Stricter cut-off values were chosen to account for the fact that this technique is often performed after an initial improvement in symptoms.

### 2.4. Safety

#### 2.4.1. Contrast Volume and Radiation Exposure Were Recorded

After embolization, all patients were monitored in the dedicated care unit for pain. They were discharged from the hospital if no complications arose. Major complications were defined as events possibly resulting in serious consequences for the patient, whereas minor complications were those not considered life-threatening. Isolated fever or pain were not deemed to be complications. Pain management and follow-up of complications (urinary or genital infection, puncture site complications, urinary or bowel dysfunction) were provided at home by a healthcare service provider during the first week after embolization. Step 1 and Step 2 analgesics, as well as anti-inflammatory drugs, were prescribed for one week following embolization. In cases where embolization of associated vulvar varices was performed, corticosteroids were also prescribed.

#### 2.4.2. Clinical and Imaging Follow-Up

All patients underwent imaging and clinical follow-up. Follow-up imaging was performed 3 months after embolization using MRI or US.

#### 2.4.3. Statistical Analysis

Descriptive statistics were used to summarize the results. Categorical variables are expressed as percentages with their 95% confidence intervals (95% CIs), calculated using the exact binomial (Clopper–Pearson) method. Continuous variables are presented as median [interquartile range, IQR]. Statistical analysis was performed using the Wilcoxon test, and a *p*-value of <0.05 was considered statistically significant (Medcalc, Version 23.1.7).

## 3. Results

### 3.1. Patients

Among the ten included patients, only one was nulliparous without endovascular embolization (patient 7). All patients experienced pelvic pain prior to treatment, with a median VAS score of 7 (6–8). Dyspareunia was also reported for all patients, with a median VAS score of 7 (5–8), as well as postcoital pain with a median VAS score of 7 (6–7). Only one patient did not report postcoital pain (patient 3).

Following endovascular embolization, a significant reduction was observed in pelvic pain (*p* = 0.002), with a median VAS score of 2 (0–3), dyspareunia (*p* = 0.03) and postcoital pain *p* = 0.004) without disappearance of symptoms. The most frequent embolized veins included the left ovarian vein in 80% of cases, the pudendal veins in 40%, and the right ovarian vein in 30%. Endovaginally guided embolization was performed on average 5 months (range 4–7 months) after endovascular embolization.

### 3.2. Procedure Safety and Technical Success

The technical effectiveness of the embolization procedures targeting the varicose veins was 100% (10/10). Local anesthesia with sedation was performed for two patients (20%) and general anesthesia for eight patients (80%). Embolization was bilateral for seven women (70%), on the right side for two women (20%), and on the left side for one woman (10%). Eight women were embolized with glue (80%) and two with Onyx^®^ (20%). The median volume of glue was 4 mL (range: 1–9). The median volume of Onyx^®^ was 4 mL (range: 3–5). The median fluoroscopy time was 5.1 min ([Q1–Q3: 3.5–7.0] range: 2.7–22.2) and the procedure duration was 55.5 min ([Q1–Q3: 48.8–71.0] range: 45.1–74.3). The median volume of iodine contrast used was 50 mL ([Q1–Q3: 40–58] range: 20–120). No complications occurred during the procedure.

### 3.3. Primary Efficacy and Outcomes

**Figure 3:**
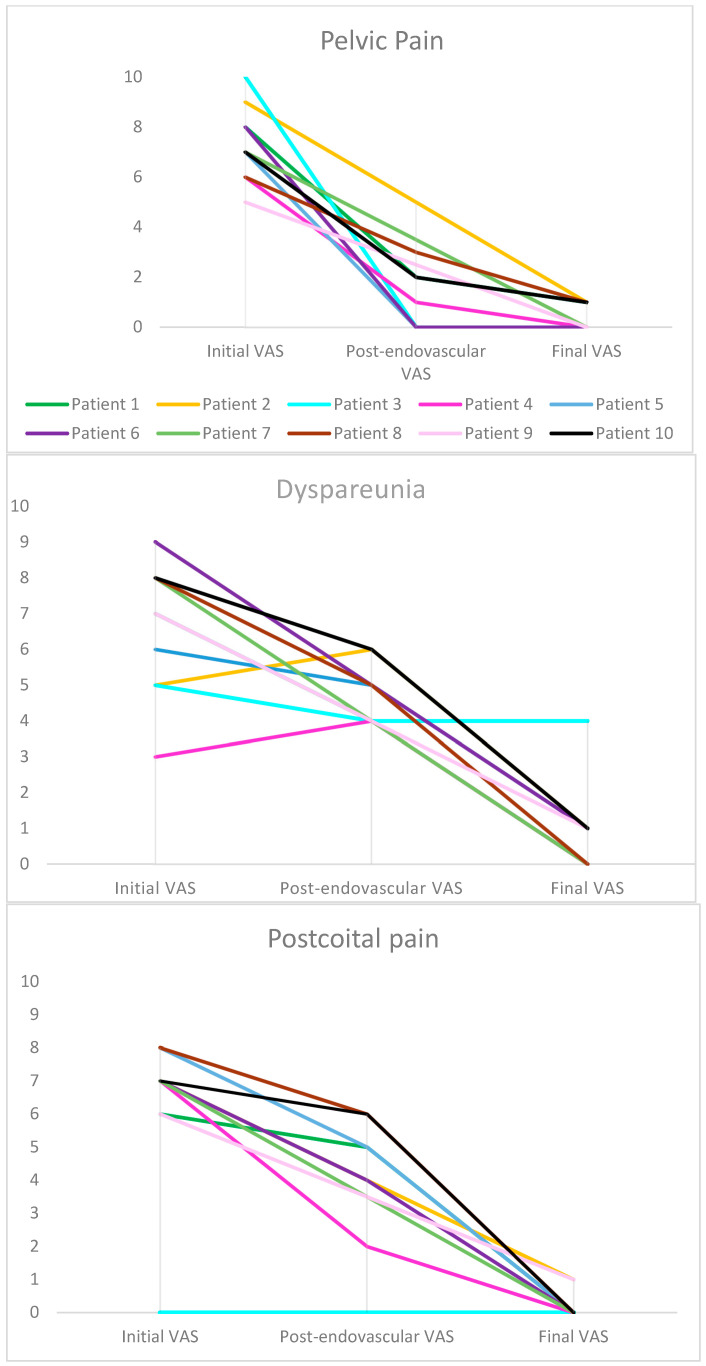
Comparison between initial Visual Analog Scale (VAS), post-endovascular VAS, and final VAS. A decrease in pain based on VAS scores was observed in all patients except for patient 3, in whom embolization by endovaginal guidance had no beneficial effect. This patient presented with a different symptom profile, notably the absence of initial postcoital pain. Each patient is represented by a different color.

**Figure 4:**
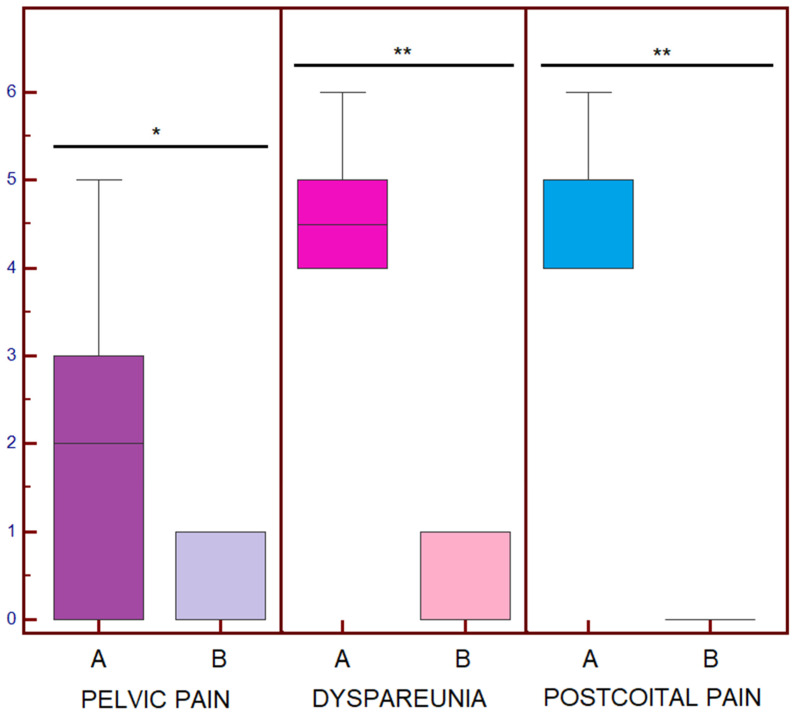
Boxplots of the post-endovascular Visual Analogue Scale (VAS) score (**A**) and final VAS score (**B**). * *p* = 0.03; ** *p* = 0.016.

The primary efficacy of the procedures was 90.0% (9/10) (95% CI: 56–100%). A significant reduction in VAS scores was observed following endovaginal intervention for dyspareunia with a median final VAS score of 1 (0–1) compared to an initial VAS score of 7 (5–8) and a post-endovascular VAS score of 5 (4–5) (*p* = 0.002 and *p* = 0.016, respectively). A significant reduction in VAS scores was observed following endovaginal intervention also for postcoital pain with a final VAS score of 0 (0–0) compared to an initial VAS score of 7 (6–7) and a post-endovascular VAS score of 5 (4–5) (*p* = 0.004 and *p* = 0.016, respectively). The difference was not interpretable in terms of chronic pelvic pain, as it was already considered treated by endovascular embolization (Figure 3 and Figure 4).

**Figure 5:**
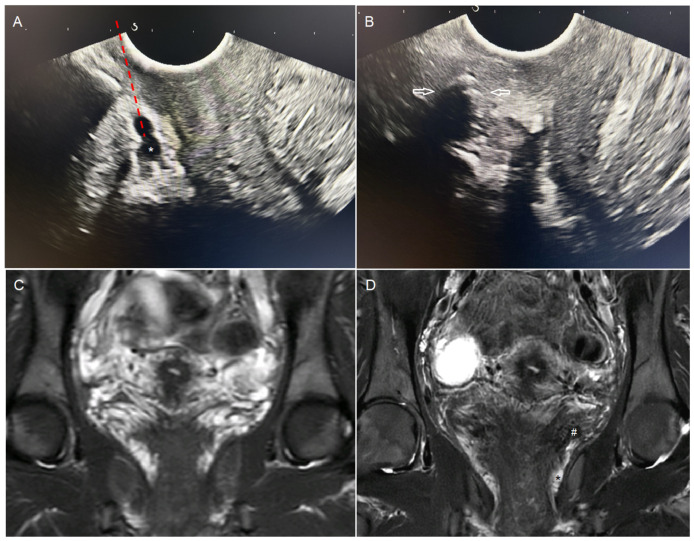
Comparison of ultrasound and MRI images before and after embolization. (**A**) Visualization of perivaginal varicosities (*) on endovaginal ultrasound. Simulated needle trajectory (---). (**B**) Same ultrasound image immediately after embolization. Attenuation artifact from the embolic agent with posterior acoustic shadowing (white arrows). (**C**) Pre-embolization MRI. Coronal T2 STIR sequence through the uterine torus and vagina. Multiple bilateral peri-uterine and perivaginal varicosities appear hyperintense. (**D**) MRI at 3 months after embolization by endovaginal guidance. Coronal T2 STIR sequence at the same level. Marked reduction in peri-uterine and perivaginal varicosities. Embolization material appears hypointense within the veins (#). Persistent dilated left perivaginal varices, though the patient was asymptomatic (*).

There were no major complication. One patient reported a minor complication consisting of transient dysuria, which resolved spontaneously in less than a week (1/10, 10%). Imaging follow-up showed a significant reduction in perivaginal varicosities in all cases (100%) (Figure 5).

## 4. Discussion

This study introduces a new approach and guidance technique for the embolization of pelvic varices.

We demonstrate that embolization of symptomatic perivaginal varices is effective, quick to perform, and seems to be safe, using a percutaneous approach under endovaginal guidance. This technique allows access to previously inaccessible veins, with the additional benefits of reduced procedure time, lower radiation exposure, and reduced contrast use. The clinical efficacy observed in the initial patient cohort is excellent, with a success rate of 90% at 3 months and no major complications reported.

The results show very low VAS scores at 3 months, which is atypical for this pathology. Indeed, the literature indicates that pain levels generally decrease over the course of a year following embolization, with an incomplete reduction at 3 months [11]. This rapid improvement is likely related to the direct occlusion of the pathological veins responsible for the pain and the use of liquid embolic agent [4,5,12]. Endovascular embolizations are often incomplete in the pelvic veins; however, the cessation of reflux leads, over a few months, to a progressive reduction in the caliber of the pelvic veins, resulting in delayed clinical improvement.

The advantage of endovaginal guidance lies in its ability to reach deeper veins. While vulvar varices have been treated for many years via percutaneous techniques using sclerosants or glue, this direct puncture approach enables embolization of afferent veins, such as the pudendal and obturator veins, but rarely allows access to the perivaginal network responsible for dyspareunia and postcoital pain [13,14]. However, even in such cases, the perivaginal network, which can be directly fed by the anterior trunk of the internal iliac veins, often remains inaccessible.

In our cohort, one patient did not benefit from embolization under endovaginal guidance. This patient initially presented with dyspareunia but no postcoital pain. It is possible that postcoital pain is a highly specific symptom related to perivaginal varicosities. Although no definitive conclusions can be drawn from such a small sample size, this finding should be considered in future studies. Indeed, postcoital pain has previously been described as one of the most discriminative symptoms of pelvic congestion syndrome, with a 94% sensitivity and a 77% specificity for the combination of ovarian point tenderness and postcoital pain [15]. Although deep dyspareunia is common among women with pelvic pain from a variety of causes, pain of venous origin is more likely to be associated with prolonged postcoital ache [16,17].

Doppler ultrasound guidance enables a targeted and personalized treatment approach tailored to the patient’s symptoms. In cases of uncertainty, an endovaginal ultrasound can be performed during consultation to confirm pain upon probe pressure [18,19]. There is, nonetheless, a learning curve with this endovaginal ultrasound-guided puncture technique, and it is recommended for radiologists already experienced with ultrasound-guided procedures, such as endovaginal drainages or prostate biopsies. Targeting varices dilated beyond 5 mm facilitates puncture, and a minimum diameter greater 3 mm is required to allow accurate targeting without compressing them with the probe. With regard to patient selection, persistence of symptoms after a first endovascular embolization represents an appropriate indication. Direct use of this technique may also be considered when vascular narrowing or obstacles are evident on pre-interventional cross-sectional imaging, making endovascular access particularly challenging.

Any liquid embolic agent can be used via the percutaneous route. This study demonstrates the successful use of glue and EVOH. It is important to consider the superficiality of the varices when selecting the embolic agent, as EVOH may cause skin staining [10]. The dilution of the glue should be adjusted according to the venous network and drainage pattern observed on antegrade phlebography, typically 1:1 or 1:2 (Glubran/Lipiodol).

The limitations of this study include the small number of patients treated, as this is an emerging technique. Most patients had undergone endovascular embolization that failed to catheterize the perivaginal varices. This subgroup represents a small proportion of patients treated for this condition, but early publication is warranted given the absence of prior research and the need to inform the scientific community about a potential therapeutic option. It may, therefore, be relevant to directly select patients who are likely to benefit from this technique, in order to perform both endovascular and endovaginal embolization during the same procedure, as was performed for one patient in the cohort. Finally, the 3-month follow-up period is likely insufficient to fully assess the risk of recurrence.

## 5. Conclusions

Embolization of perivaginal varices using an endovaginal approach is an effective alternative to the endovascular route. A prospective study with a larger cohort and a longer follow-up is required to confirm the efficacy and safety of this novel technique. This approach also represents a further step toward personalized medicine in interventional radiology, with adaptation of the access route and guidance technique to the patient’s symptoms.

## Figures and Tables

**Table 1:** Patient characteristics.

N	Age (Years)	G-P	Duration Pain (Years)	Initial VAS	Embolized Veins *	Post-Endovascular VAS	Final VAS	Perivaginal Veins Size (mm)
**1**	33	G2P2	8	8-7-6	LOV ROV RPV	2-4-5	1-0-0	4
**2**	37	G3P3	7	9-5-7	LOV ObV RVV	5-6-4	2-1-1	3
**3**	39	G6P4	3	10-5-0	LOV UV PV	0-4-0	0-4-0	2
**4**	45	G3P2	15	6-3-7	LOV LVV	1-4-2	0-0-0	5
**5**	32	G2P2	1	7-6-8	LOV ROV LUV	0-5-5	0-1-0	4
**6**	35	G1P1	3	8-9-7	LUV LVV	0-5-4	0-1-0	4
**7**	23	G0P0	3	7-8-7	NA	N/A	0-0-0	3
**8**	36	G2P2	5	6-8-8	LOV LPV ObV	3-5-6	1-0-0	3
**9**	40	G4P2	8	5-7-6	LOV	N/A	0-1-1	4
**10**	32	G2P1	5	7-8-7	LOV ROV LUV PV	2-6-6	1-1-0	3

G-P: Gravida-Para; VAS: Visual Analog Scale; LOV: left ovarian vein; ROV: right ovarian vein; RPV: right pudendal vein; LPV: left pudendal vein; RVV: right vaginal vein; UV: uterine veins; PV: pudendal veins; LVV: left vaginal vein; LUV: left uterine vein; ObV: obturator veins; VAS: pelvic pain–dyspareunia–postcoital pain; N/A: Not Applicable. * By endovascular approach.

## Data Availability

The original contributions presented in this study are included in the article. Further inquiries can be directed to the corresponding author.

## Abbreviations

The following abbreviations are used in this manuscript:

VAS	Visual Analogue Scale
EVOH	Ethylene vinyl alcohol copolymer
DMSO	Dimethyl sulfoxide
